# A novel serine hydroxymethyltransferase from *Arthrobacter nicotianae*: characterization and improving catalytic efficiency by rational design

**DOI:** 10.1186/s12896-014-0093-9

**Published:** 2014-11-14

**Authors:** Wei Jiang, Lin Chen, Nan Hu, Shaohui Yuan, Bin Li, Ziduo Liu

**Affiliations:** State Key Laboratory of Agricultural Microbiology, College of Life Science and Technology, Huazhong Agricultural University, Wuhan, 430070 P. R. China; College of Biotechnology and Pharmaceutical Engineering, Nanjing Tech University, Nanjing, 211800 P. R. China

**Keywords:** *Arthrobacter nicotianae*, SHMT, Characterization, Site-directed mutagenesis, Catalytic efficiency

## Abstract

**Background:**

Serine hydroxymethyltransferase (SHMT) is the key enzyme in L-serine enzymatic production, suggesting the importance of obtaining a SHMT with high activity.

**Results:**

Here, a novel SHMT gene, *glyA*, was obtained through degenerate oligonucleotide-primed PCR and encoded a novel SHMT with 54.3% similarity to the known SHMT from *Escherichia coli*. The obtained protein *An*SHMT showed the optimal activity at 40°C and pH 7.5, and was more stable in weakly alkali conditions (pH 6.5-8.5) than *Hyphomicrobium methylovorum*’s SHMT (pH 6.0-7.5), In order to improve the catalytic efficiency of the wild type, the site-directed mutagenesis based on sequences alignment and bioinformatics prediction, was used and the catalytic efficiency of the mutant I249L was found to be 2.78-fold higher than that of the wild-type, with the replacement of isoleucine by leucine at the 249 position.

**Conclusions:**

This research provides useful information about the interesting site, and the application of DOP-PCR in cloning a novel *glyA* gene.

## Background

There have been only a few reports about the enzymatic characterization and the change of the enzymatic properties of SHMT by protein engineering [1,2]. Currently, L-serine production mainly relies on enzymatic conversion from glycine precursor plus a C_1_ compound [3], and the key enzyme in L-serine enzymatic conversion is SHMT. The SHMT is coded by the *glyA* genes and act as the first enzyme in the assimilation of C_1_ compounds through the addition of formaldehyde to glycine, producing the principal intermediate in the pathway, serine [4]. Therefore, it is necessary to obtain a SHMT with high activity of some new microorganisms and improve the catalytic efficiency through in vitro directed evolution.

SHMT (EC 2.1.2.1), a member of the α-class of pyridoxal phosphate enzymes, catalyzes the reversible interconversion of serine and glycine, changes the chemical bonding at the C^α^-C^β^ bond of the serine side-chain mediated by the pyridoxal phosphate cofactor [5]. The crystal structures of SHMT from the human and rabbit confirm that SHMT belongs to the α-family of enzymes and shares a similar tertiary fold and mechanism [6,7]. SHMT is ubiquitous, highly conserved PLP-dependent enzyme with tetrahydrofolate (THFA) as the C_1_ acceptor [8], purified from the animals [9,10], plants [11] and bacteria [12]. SHMT is widely used in the synthesis of the serine using glycine and formaldehyde, while the activity of the wild-type is not sufficient for industrial production [2].

The degenerate oligonucleotide-primed PCR (DOP-PCR) has been applied to the characterization of abnormal chromosomes and also in the cloning of new markers for specific chromosome regions [13]. Based on unknown genome information circumstances, using DOP-PCR to obtain *glyA* (encoding SHMT) can overcome the shortcomings of existing methods, such as cDNA library [14,15], southern-blot hybridization [16], direct PCR amplification according to the known genome information [17,18], and shotgun technology [19], for their waste time and material under the same conditions. Protein engineers redesign proteins in order to improve their biomedical or industrial utility [20]. Although the crystal structures of SHMT from the human, rabbit and *Escherichia coli* (*E. coli*) are reported, little is known at present about the structure-function relationship among these enzymes [5-7]. And the exact active-site residues have never been confirmed using kinetic analyses of the wild-type and variant enzymes. Structure-based site-directed mutagenesis is usually applied to produce variants with dramatically improved specificities and the residues in or near the active site tend to be chosen as their special roles in the activity of the enzymes [21-23].

The objectives of the current study were to (i) clone the glyA gene from *Arthrobacter nicotianae* (*A. nicotianae*) using degenerate primers, (ii) study characteristics of recombinant SHMT by isolating the enzyme with high activity and (iii) improve its catalytic efficiency by site-directed mutagenesis.

## Results

### Cloning of *glyA* gene

**Figure 1:**
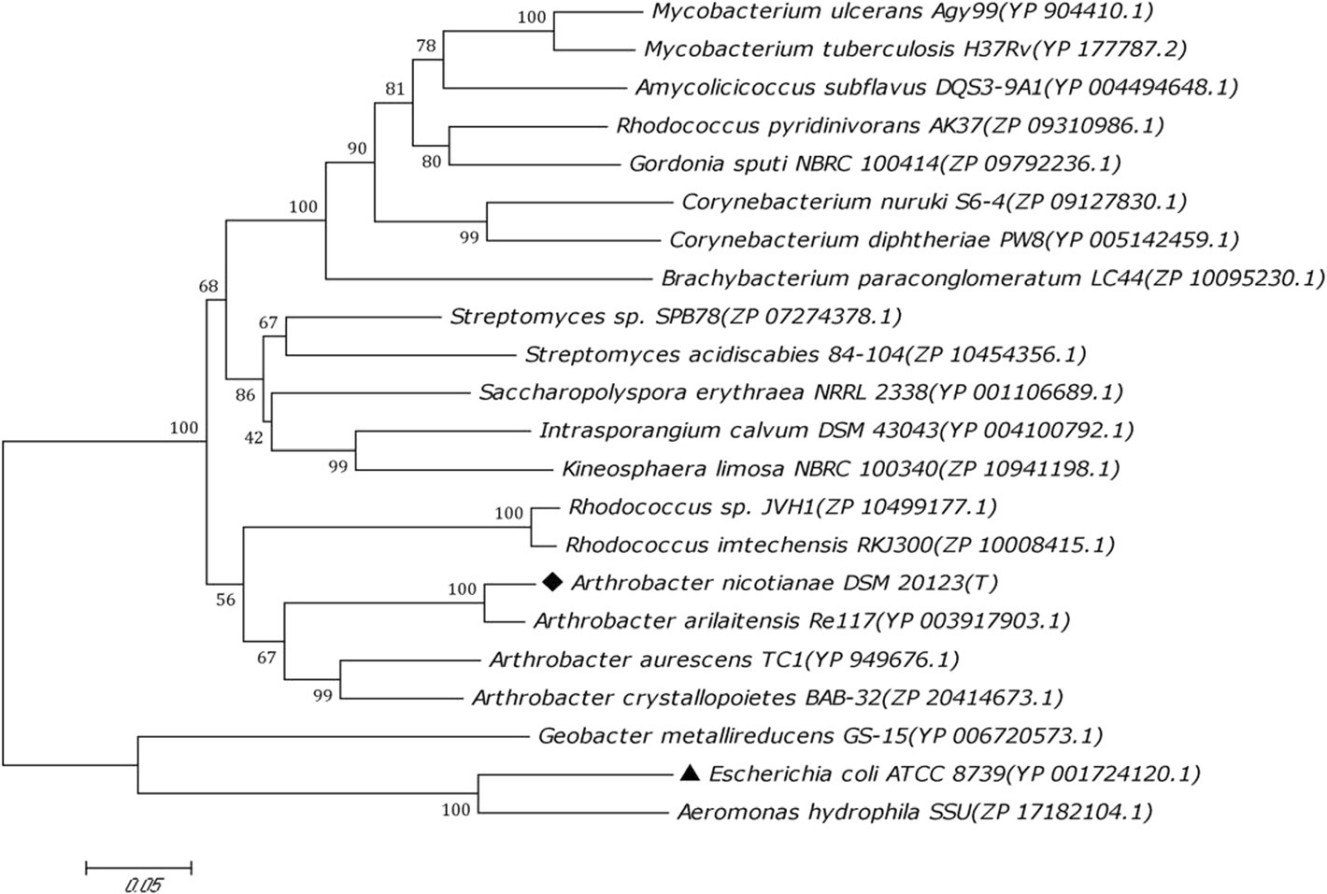
**Phylogenetic tree of the AnSHMT.** The phylogenetic tree was established using the program MEGA 5.05. The SHMT protein sequences were obtained from GenBank and PDB (http://www.rcsb.org/pdb/home/home.do), except for AnSHMT.

Using degenerate primers, a fragment was obtained by DOP-PCR with the genomic DNA of *A. nicotianae* as the template. The open reading frame (ORF) of the *glyA* (1323 bp, GenBank: KF359496) fragment encoded a polypeptide of 440 amino acids, with a deduced molecular mass of 47.3 kDa. The phylogenetic analyses of the SHMT sequences produced a tree to further verify the evolutionary relationship among *An*SHMT and other known SHMTs, indicating that the *An*SHMT shared 54.3% amino acid identity with the known SHMT from *E. coli* (Figure 1).

### Bioinformatic analysis of the amino acid sequence

**Figure 2:**
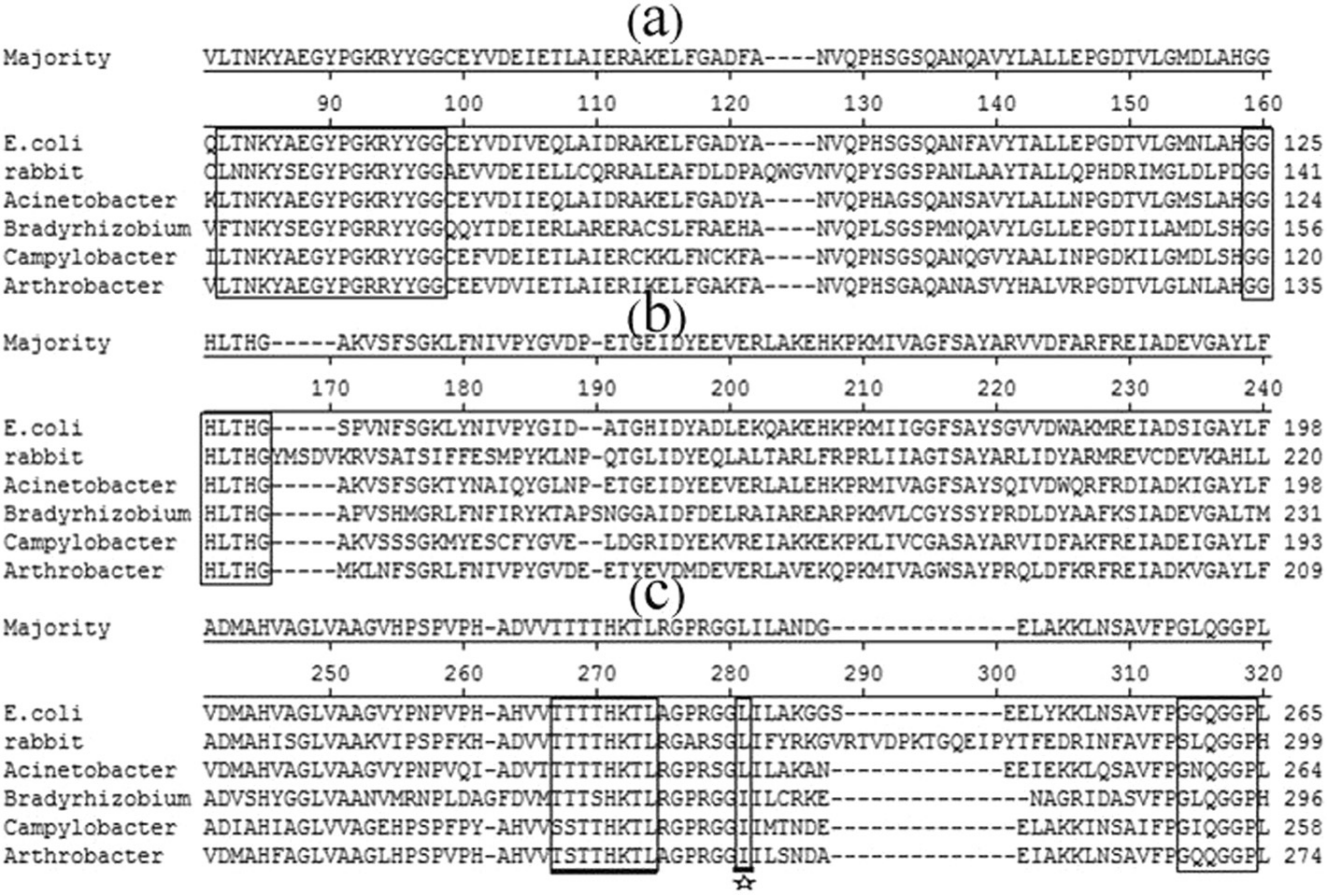
**Multiple sequence comparison by ClustalW. (a)** a part of the sequence alignment result (81-160 sites); **(b)** a part of the sequence alignment result (161-240 sites); **(c)** a part of the sequence alignment result (241-320 sites). Open boxes indicate the four highly conserved regions and the conserved active site is underlined. The site-directed mutagenesis site is underlined and is marked by five-pointed star. The amino acid sequences were obtained from NCBI database (http://www.ncbi.nlm.nih.gov/).

**Figure 3:**
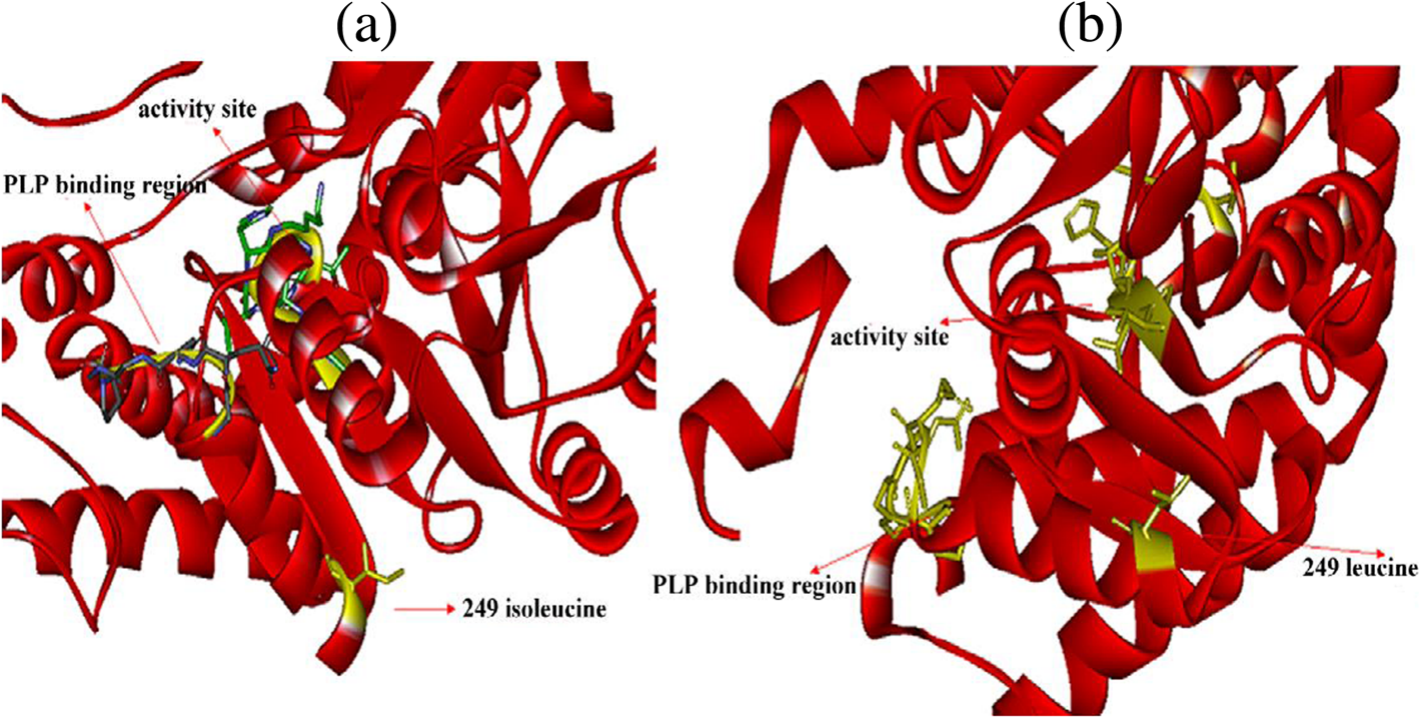
**Modeled three**-**dimensional structure of the** ***An*** **SHMT. (a)** the three-dimensional structure of the wild type enzyme; **(b)** the three-dimensional structure of the mutational enzyme. The catalytic site, PLP binding region and the site of site-directed mutagenesis were indicated on the three-dimensional structure, respectively. The three-dimensional structure was generated using the Swiss-Pdb viewer.

Multiple sequence comparisons were performed by Clustal W Method, indicating that the conserved amino acid sequences and motifs are critical for the active site of SHMT (Figure 2). The highly conserved active site in all known SHMT enzymes T/ST/STTHKT/SL was also found in *An*SHMT (235–242) in the form of TSTTHKTL (Figure 2c), the putative active site [5,14,24]. The site of the site-directed mutagenesis underlined and marked by a five-pointed star was the close proximity to the highly conserved active site and the putative active site (TSTTHKTL) (Figure 2c). The deduced active site structure (TSTTHKTL) and the position of the mutant (249 isoleucine) were briefly presented the three-dimensional structure of *An*SHMT (Figure 3).

Based on multiple sequence comparisons, two well-known conserved sequences LTNKYAEGYPGRRYYGG (61–77) and GGHLTHG (134–140) (Figure 2a and 2b) were also detected in *An*SHMT [25]. The glycine rich region GQQGGP (268–273) (Figure 2c), was highly conserved sequence, significant homologous sequence, and proposed to be essential for PLP binding [24], the region of which was indicated on the three-dimensional structure of *An*SHMT (Figure 3).

### Site-directed mutagenesis

A leucine residue was introduced into the 249 position using site-directed mutagenesis and conformed, followed by the test of its effects on enzymatic activity using purification I249L-SHMT.

### Expression, purification and activity of the *An*SHMT and the mutant

**Figure 4:**
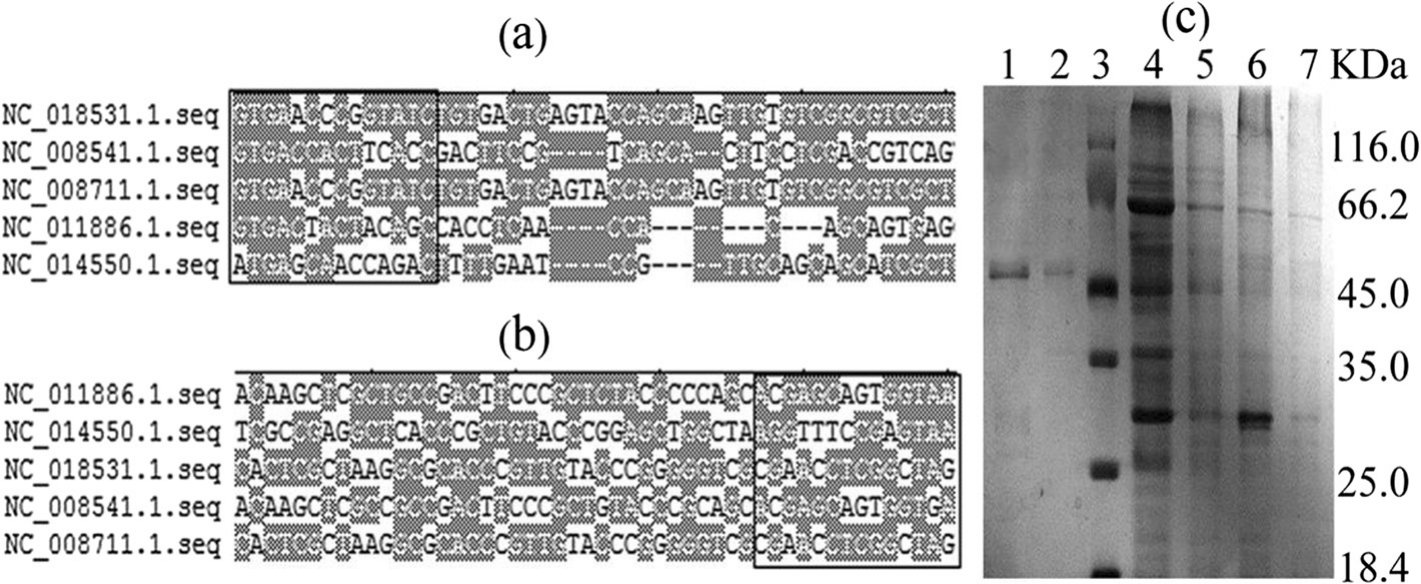
**Design of both end degenerate primers and 12%** **SDS**-**PAGE analysis of the purified proteins.** **(a and b)** The *glyA* gene sequences were obtained from NCBI database and belonged to the genus of *Arthrobacter* (GI: NC_008541; NC_008711.1; NC_011886.1; NC_014550.1; NC_018531.1). Degenerate primers were designed by the regions in the boxes. **(c)** 12% SDS-PAGE analysis of the purified proteins. The bands in the ellipses show GST (glutathione-S-transferase) and fusion protein (*An*SHMT and GST) respectively. Lane 1: purified I249L without GST. Lane 2: purified *An*SHMT (the wild-type enzyme) without GST. Lane 3: protein marker. Lane 4: recombinant bacterium (harboring pGEX-6P-*glyA*) induced by IPTG. Lane 5: recombinant bacterium (harboring pGEX-6P-*glyA*) non-induced by 0.1 mM IPTG. Lane 6: bacterium (harboring pGEX-6p-1) induced by IPTG. Lane 7: bacterium (harboring pGEX-6p-1) non-induced by 0.1 mM IPTG. The protein molecular weight ladder is Unstained Protein Molecular Weight Marker (Fermentas, Canada).

The *glyA* was cloned into the vector pGEX-6p-1 and expressed in DE3. The induced and non-induced recombinant bacteria (harboring pGEX-6p-*glyA*) and the induced control bacterium (harboring empty pGEX-6p-1 vector) were examined with SDS-PAGE (Figure 4c). After purification with Glutathione Sepharose 4B and digestion with 3C protease, the recombinant *An*SHMT and I249L-SHMT were harvested and resolved to a single band, with the purified *An*SHMT exhibiting the expected molecular mass (47.3 kDa) (Figure 4c).

The steady-state kinetic parameters of the wild-type enzyme and the I249L-SHMT were measured at 40°C at a DL-3-phenylserine concentration from 0.1–16.3 mg/ml (Table 1). Mutant I249L showed a 7% increase in *Km* and 2.97-fold increase in *k*_*cat*_, resulting in approximately 2.78-fold increase in (*k*_*cat*_/*Km*). The results indicated that the catalytic efficiency was considerably improved by replacing the isoleucine with leucine at the 249 position.

**Table 1:** **Steady**-**state kinetic parameters for the wild**-**type** ***An*** **SHMT and the mutant**

**Enzyme**	***Km*** **(mM)**	***k***_***cat***_ **(min** ^**−1**^ **)**	***k***_***cat***_ **/** ***Km*** **(min** ^**−1**^ **M** ^**−1**^)
WT	57.86 ± 0.07	96.02 ± 0.12	1.66 × 10^3^
I249L	61.95 ± 0.13	285.3 ± 0.15	4.61 × 10^3^

*Note*. The data are the average of three replicates.

### Effects of temperature and pH on enzyme activity

**Figure 5:**
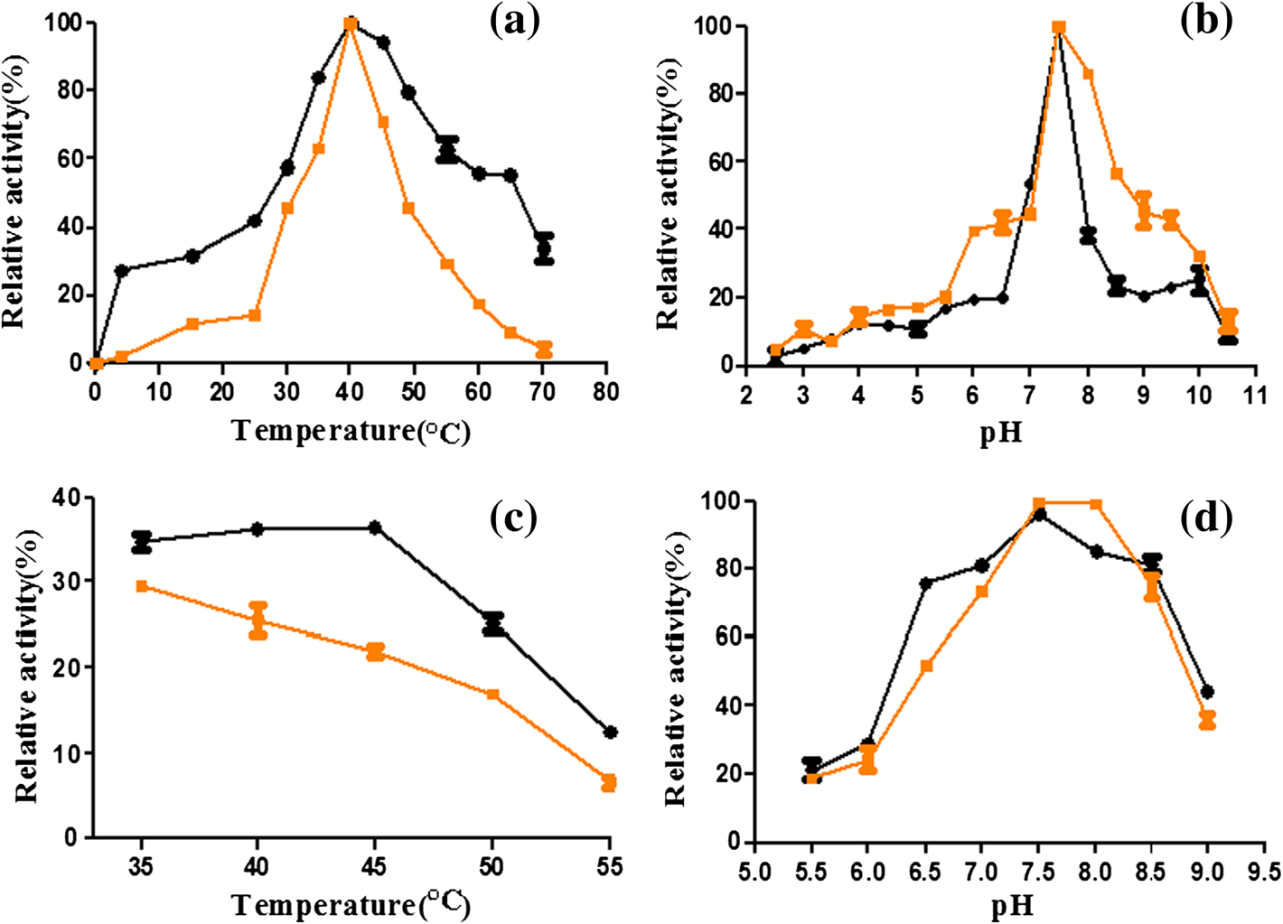
**Effects of temperature and pH on activity. (a)** Effect of temperature on the activity of the *An*SHMT and the I249L-SHMT. The optimal temperature was determined by measuring the activity at temperatures from 0 to 70°C. The maximal activity was taken as 100%. **(b)** Effect of pH on the activity of the enzymes. The activity was measured over a pH values ranging from 2.5 to 10.5, and the maximal activity was taken as 100%. **(c)** Effect of temperature on the stability of the *An*SHMT and the I249L-SHMT. At the optimal pH 7.5, the purified enzyme was pre-treated at a different temperature for 1 h. The activity of the enzyme without pre-incubation was defined as 100%. **(d)** The pH stability of the enzymes was determined by incubating the enzymes at a different pH at 4°C for 24 h. Then assays were conducted in the standard conditions and the enzyme activity without pre-treatment was taken as 100%. Error bars represent the standard deviation. Black circles represent the *An*SHMT (●), and black squares represent the I249L-SHMT (■).

The maximal activity of the *An*SHMT was observed at 40°C, retaining over 50% of the maximal activity at temperatures from 30°C to 65°C (Figure 5a). The I249L-SHMT diaplayed the same optimum temperature (40°C) with the *An*SHMT (Figure 5a). After 1 h incubation under pH 7.5 (Figure 5c), the *An*SHMT retained over 35% of its maximal activity from 35°C to 45°C, but less than 15% at 55°C.

The *An*SHMT exhibited the optimal activity at pH 7.5, and was sensitive in low pH buffers, displaying less than 20% of its maximal activity at pH 5.5 and nearly no activity was detected below pH 2.5 (Figure 5b). Similarly, the I249L-SHMT also showed the maximal activity at pH 7.5, and retained over 85% of its maximal activity at pH 8.0 (Figure 5b). Without any stabilizer, the purified *An*SHMT showed significant stability under weakly acidic and alkaline environment (pH 6.5-8.5), retaining over 80% of the maximum activity over a pH range from 7.0 to 8.5 for 24 h at 4°C, and more than 70% of the maximal activity at pH 6.5. However, the *An*SHMT exhibited a rapid decrease in activity at pH 5.5 (Figure 5d).

The effects of different metal ions or chemical reagents on *An*SHMT are presented in Table 2. The enzyme activated by Mg^2+^, Ba^2+^ and EDTA, but could be strongly inhibited by Hg^2+^, Cu^2+^, Zn^2+^ and Fe^2+^, and slightly inhibited by Co^2+^, Mn^2+^, DTT, and CO(NH_2_)_2_. Besides, the enzyme was not significantly influenced in activity by NH_4_^+^, Sl^2+^, Ca^2+^, Pb^2+^, SDS and CTAB.

**Table 2:** **Effects of metal ions and chemical reagents on the activity of purified** ***An*** **SHMT** ^**a**^

**Reagents**	**Concentration** **(mM or %)**	**Relative activity (%)**
control	0	100%
Hg^2+^	1	- ^b^
Co^2+^	1	60.03 ± 0.27 c
K^+^	1	100.44 ± 0.37
Na^+^	1	100.62 ± 0.30
NH_4_^+^	1	95.12 ± 0.58
Sl^2+^	1	94.44 ± 0.83
Mg^2+^	1	108.38 ± 0.85
Ca^2+^	1	91.38 ± 0.11
Ba^2+^	1	102.58 ± 0.33
Cu^2+^	1	8.95 ± 0.36
Zn^2+^	1	2.91 ± 0.11
Mn^2+^	1	60.81 ± 0.28
Fe^2+^	1	- ^b^
Pb^2+^	1	98.90 ± 0.32
CO(NH_2_)_2_	1	60.59 ± 0.45
DTT	1	59.29 ± 0.08
CTAB	0.3%	97.05 ± 0.04
EDTA	1%	101.25 ± 0.65
SDS	5%	- ^b^

^a^: all assays were performed in the standard conditions and the activity measured without additional reagents and ions was taken as 100%. The data are the average of three replicates; ^b^: unmeasured data; ^c^: Relative activity ± the standard deviation.

## Discussion

The taxonomy of the bacterium *A. nicotianae* (ATCC 15236) was studied in 1982 [26]. While no paper on the characterization of the SHMT from *Arthrobacter* was reported. The full-length of the *glyA* gene from *A. nicotianae* was obtained by DOP-PCR [13] with moderate modifications, which represents a rapid, efficient, and species-independent technique for general DNA amplification. To the best of our knowledge, this is the first report on the application of this technique to clone the *glyA* gene, suggesting that its potential application in the cloning of other *glyA* genes. The *glyA* encoding a novel SHMT shared 54.3%, 53.6%, 50.9%, 41.2%, and 40.3% similarities to the known enzymes from *E. coli*, *H. methylovorum* GM2, *C. jejuni*, *B. japonicum*, and rabbit liver mitochondria, respectively.

Currently, L-serine is mainly produced through cellular or enzymatic conversion from the precursor glycine plus a C_1_ compound, in which SHMT is the key enzyme [3,4]. *E. coli* SHMT forms a tight homodimer similar to that of AAT and other α-PLP enzymes, with the active site at the interface of the two monomers [5]. Directed evolution is a technique that can overcome the limitations of natural enzymes used as biocatalysts, for this technique does not only rely on a detailed understanding of the relationship between enzyme structure and function, but on the simple powerful Darwinian principles of mutation and selection [27]. By targeting mutations to certain amino acids, we can map the enzyme active site, investigate mechanisms, and study structure-function relationships [28]. The site-directed mutagenesis methods are usually used to generate cloned DNAs with modified sequences to examine the importance of specific residues in the protein structure and function [29]. Therefore, studying the SHMT enzymatic properties and improving the catalytic efficiency can be available for insight into the industrial production of L-serine and further research.

To the best of our knowledge, this is the first report of the characterization of the SHMT which comes from *Arthrobacter*. Unlike *Hyphomicrobium methylovorum*’s SHMT (37°C) [4], the *An*SHMT exhibited the highest activity at 40°C in the assays of the enzymatic characteristics, and retained 84% and 94.2% of the maximal activity at 35°C and 45°C, respectively, and less than 40% of the activity after 1 h pre-treatment at 35°C and 45°C. Without any stabilizer, the *An*SHMT displayed higher pH stability profiles (pH 6.5-8.5) than that from *Hyphomicrobium methylovorum* (pH 6.0 and 7.5) under alkaline environment. The I249L-SHMT showed nearly no loss in activity after 24 h incubation under pH 8.0, while the *An*SHMT retained about 85% of the maximum activity. The *An*SHMT was not affected by EDTA, indicating that the metal ions were not appropriate to the enzyme. Additionally, the loss of activity of the enzyme in the presence of Hg^2+^ might be attributed to the interaction with thiol groups of Cys residue(s), dry residue(s), or carboxyl group(s) of amino acid(s) [30].

The replacement of isoleucine with leucine at the 249 position resulted in a 2.78-fold increase of the catalytic efficiency (*k*_*cat*_/*Km*) in the mutant I249L over the wide type, namely a 7% increase in *K*_m_ and a 2.97-fold increase in *k*_*cat*_ indicating that the 249 site is vital to improve the enzyme activity. The increase of the 7% for the *K*_m_ may be due to the decreased of enzyme and substrate affinity with the replacement of isoleucine by leucine at the 249 position. The results of the site-directed mutagenesis performed by the sequences alignment and bioinformatics analysis indicate that the 249 site is vital to improve the enzyme activity. The improvement of the catalytic efficiency could be attributed to four factors: (i) based on the model structures, the mutant is too far away from the substrate to participate in direct interaction, resulting in no mutation in the catalytic active site (Figure 3), which could be due to the possibility that the occurrence of most mutations in the active site would inactivate the enzyme, because SHMT is one of the most highly conserved proteins, and the mutations likely exert their effects by changing the conformation of the active site rather than by interacting directly with substrates [2]; (ii) the three-dimensional structure (Figure 3) of *An*SHMT shows that the isoleucine (249 site) is probably located at the end of an active channel and the PLP binding region at the other end of the active channel, so the change of the 249 site residue has no effect on the PLP binding progress; (iii) when PLP or other molecules bind the enzyme, the steric hindrance may be reduced by the residue and is altered by leucine; and (iv) as the relative abundance of isoleucine and leucine are 4.4 and 7.8, respectively, more steric hindrance is produced by the binding of the substrate to the enzymatic molecules through the cavity of the former. Further exploration concerning the structure-activity relationship of SHMT is necessary owing to the limited information about the relationship [5-7] and the interesting site would be useful for these work.

## Conclusion

The *An*SHMT in the present study is a novel one from *A. nicotianae*, sharing 54.3% similarity to the known SHMT from *E. coli*. The enzyme showed better stability under weakly alkali conditions (pH 6.5-8.5) than *Hyphomicrobium methylovorum*’s SHMT (pH 6.0 and 7.5), and exhibited the optimal activity at pH 7.5 and 40°C. Through site-directed mutagenesis, the catalytic efficiency (*k*_*cat*_/*Km*) of the mutant I249L was 2.78-fold higher than that of wide type. These characteristics provide useful information about the interesting site, and the application of DOP-PCR in cloning a novel *glyA* gene.

## Methods

### Reagents, bacterial strains, vectors and cultivation conditions

The restriction enzymes, T4 DNA ligase, *Taq* DNA polymerase, pMD18-T vectors, molecular weight marker, gel extraction kit and the DNA purification Kit used in the research were all from purchased from TaKaRa Co. (Dalian, China) and Qiagen Co. (Germany). The *Pfu* DNA polymerase, PreScission protease and the GST · Bind Resin were purchased from TransGen Co. (China), GE Healthcare Co. and Merck Co. (Germany), respectively. All oligonucleotide primers (Table 3) and fragments were synthesized and sequence by GenScript Co. (Nanjing, China). Unless otherwise stated, all the other chemicals used in this study arose out of analytical grade and purchased from Sinopharm Chemical Reagent (Wuhan, China).

**Table 3:** **Primers used for plasmid construction and the site**-**directed mutagenesis**

**Primers**	**5**′ **to 3**′	**Purpose or references**
DP-F	RTGAMCMCKNYATC	This study
DP-R	YTACCCSWGSTCSK	This study
An--F	CGGGATCC**ATG**AGCAACCAGACTTTTGAATC	This study
An--R	CGGAATTC**CTA**CTCGGAAACCTTTGGCAGGT	This study
I249L-F	CCGCGTGGCGGCCTGATTCTGTCGA	This study
I249L-R	CAGGCCGCCACGCGGACCAGCCAAC	This study

IUPAC ambiguity codes: M = A/C, R = A/G, W = A/T, S = G/C, Y = C/T, K = G/T and N = A/G/C/T. I indicates inosine. Restriction sites *Bam*H1 and *Eco*RI in primers *An*--F/R and the mutated site are underlined; Start and stop codons are marked in bold; site-directed mutagenesis site is underline in I249L-F and I249L-R.

The SHMT screening medium [31], consisting of 125 ml/L methanol, 5 g/L NaCl and 5 g/L glycine, was ready to detect the enzyme activity of the bacterium. *A. nicotianae*, with high SHMT activity used in this study, was isolated from the Nanhu-Lake (Wuhan, China) and preserved in our laboratory and was cultivated in Luria-Bertani (LB) medium at 37°C. *E. coli* DH5a and *E. coli* BL21 (DE3) strains were cultured in Luria–Bertani (LB) medium containing ampicillin (100 μg/mL) and used as cloning and expression hosts, respectively. Plasmid pGEX-6P-1 was utilized for the preparation of the mutant and the purification of *An*SHMT and its mutant. The submitted work does not contain any experiments using animals or humans.

### Gene cloning

The full ORF of *glyA* from *A. nicotianae* was obtained by DOP-PCR using both end degenerate primers with the following program: (i) 94°C for 5 min, (ii) 30 cycles of 94°C for 30 s, 44-48°C for 30 s, and 72°C for 1 min 30 s, and (iii) 72°C for 10 min, DP-F and DP-R (Table 3), which were designed according to the result of the multiple sequence alignment (Figure 4a and 4b) between *glyA* gene sequences and the genus of *Arthrobacter* obtained from the NCBI database. After amplification, the PCR product was purified by agarose gel electrophoresis, and was cloned into PMD18-T vector and sequenced. After that, the *glyA* was obtained from the chromosome DNA of *A. nicotianae* by PCR using the forward and reverse primers (An-F and An-R, Table 3) under the following conditions: 1 cycle at 94°C for 5 min; 30 cycles at 94°C for 30 s, 52°C for 30 s and 72°C for 1 min 30 s; and 1 final additional cycle at 72°C for 10 min. After double-digestion with *Bam*HI and *EcoR*I, the resulting PCR product was gel-purified and cloned into the *Bam*HI-*EcoR*I sites of pGEX-6p-1, generating pGEX-6p-*AnglyA* containing the glutathione-S-transferase (GST) tag. The construction accuracy was confirmed by sequencing at Nanjing GenScript Company.

### Site-directed mutagenesis

The position of the site-directed mutagenesis was selected founded on the result of the multiple sequence alignment of SHMTs (Figure 2). The amino acid sequences which were reported to belong to *E. coli*, rabbit liver mitochondria, *A.radioresistens*, *B. japonicum*, *C. jejuni*, and *A. nicotianae*, respectively. The distinct amino acid residues close to activity site were observed and assumed to produce a negative effect on enzymatic activity [2]. As shown in Figure 2, an interesting phenomenon was detected in the site underlined and marked by a five-pointed star, indicating that it was isoleucine rather than leucine close to the sequence TSTTHKTL (Figure 2c), which was highly conserved in all known SHMT proteins [5,14,24].

The site-directed mutagenesis was done by PCR using the plasmid pGEX-6p-*AnglyA* as the template and primers designed from pairs of complementary oligonucleotides containing the desired mutant (Table 3). The PCR mixture (50 μl) was composed of a PCR buffer, 10 ng template pGEX-6p-*AnglyA*, 0.2 μM each oligonucleotide, 0.6 μM dNTP and 1 unit of FAST *Pfu* DNA polymerase (TransGen, China). The PCR was performed in the following three steps: (i) 97°C for 2 min; (ii) 20 cycles of 95°C for 20 s, 54°C for 20 s, and 72°C for 3 min 20 s; and (iii) 72°C for 7 min with the I249L-F and I249L-R (Table 3). The PCR product was gel-purified, treated with DpnI to eliminate methylated ancestral template through an overnight incubation at 37°C, and then transformed into *E. coli* DH5α competent cells. The recombinant plasmids were checked by DNA sequencing, and the successfully introduced desired mutation was designated as pGEX-6p-I249L.

### Expression and purification of wild-type *An*SHMT and mutant enzyme

The recombinant plasmids pGEX-6p-*AnglyA* and pGEX-6p-I249L were transformed into DE3 for the SHMT expression. DE3 strain harboring the recombinant plasmid was inoculated into LB liquid medium with ampicillin (100 μg/mL) and incubated at 37°C overnight. Subsequently, the mixture was transferred into fresh LB liquid medium (1:100 dilution) containing ampicillin (100 μg/mL) and cultured at 37°C for 3–4 h. When the cells reached an optical density of 0.6-0.8 at 600 nm, protein expression was induced by adding IPTG to a final concentration of 0.1 mM and the mixture was incubated at 18°C for 12 h. After that, the cells were harvested and disrupted with High Pressure Homogenizer (NS100IL 2 K, Niro Soavi, Germany). Finally, the SHMTs were purified by glutathione-S-transferase (GST) Gene Fusion System (GE Healthcare, Sweden).

### Measurement of SHMT activity

Standard enzyme activity of phenylserine degradation was measured with DL-3-phenylserine as substrate as previously described by Lee and Hsiao [32] with moderate modifications. Briefly, standard enzyme activity assay was performed by adding moderate purification enzyme into the 1 mL reaction system at pH 7.8 with sodium phosphate buffer containing 50 mM DL-3-phenylserine, 50 μM PLP, 1 mM Na_2_EDTA (ethylene diamine tetraacetic acid) and 25 mM sodium sulfate. When cells were used in the reaction system, 0.03% (w/v) cetyltrimethyl ammonium bromide (CTAB) was added. The reaction was conducted for at 30°C, and 1 h later, and the production of benzaldehyde was measured by its strong absorbance at 279 nm [2]. One unit of SHMT activity was defined as the amount of enzyme that released 1 μmole benzaldehyde per hour (benzaldehyde as standard). Specific activity was reported in units/mg protein.

### Biochemical characterization of the enzyme

The optimal pH of SHMT was determined at 30°C in different buffers at pH 2.5-10.5, namely 0.2 M Na_2_HPO_4_/0.1 M citric acid buffer (pH 2.5–8.0) and 50 mM glycine–NaOH buffer (pH 8.0–10.5). The optimum temperature for the enzyme was measured by performing the SHMT activity assay for 1 h at temperatures from 0 to 70°C under pH 7.5. The thermal stability of the enzyme was determined under the optimal pH by pre-incubating the enzyme at temperatures from 35°C to 55°C for 60 min and then the residual activity was measured as described above. To determine the pH stability of the SHMT, the recombinant SHMT was incubated at 4°C for 24 h in different buffer systems (pH 5.5–9.0), and the residual activity was measured under standard assay conditions. The biochemical characterization of the I249L-SHMT was performed with the same methods.

Effects of metal ions and other chemical compounds on the enzyme activity were determined in the standard reaction system at 40°C for 1 h at pH 7.5.

### Computer model generation

The position of site-directed mutagenesis was indicated on the three-dimensional structure of *An*SHMT, which was constructed from the known x-ray structure of *Burkholderia Pseudomallei Mycobacterium Tuberculosis T.Th*.Hb8 (PDB entry 3H7F, 2DKJ and 3ECD) using Swiss-Model, a knowledge-based protein modeling tool [33]. Furthermore, the PLP binding region and the active site were marked on the three-dimensional structure.
